# Impression Formation in the Human Infant Brain

**DOI:** 10.1093/texcom/tgaa070

**Published:** 2020-09-29

**Authors:** Kathleen M Krol, Tobias Grossmann

**Affiliations:** Department of Psychology, University of Virginia, Charlottesville, VA 22903, USA; Max Planck Institute for Human Cognitive and Brain Sciences, Leipzig 04275, Germany; Department of Psychology, University of Virginia, Charlottesville, VA 22903, USA; Max Planck Institute for Human Cognitive and Brain Sciences, Leipzig 04275, Germany; 1 Department of Psychology, University of Virginia, Charlottesville, VA 22903, USA; 2 Max Planck Institute for Human Cognitive and Brain Sciences, Leipzig 04275, Germany

**Keywords:** emotion, fNIRS, impression formation, infancy, mPFC

## Abstract

Forming an impression of another person is an essential aspect of human social cognition linked to medial prefrontal cortex (mPFC) function in adults. The current study examined the neurodevelopmental origins of impression formation by testing the hypothesis that infants rely on processes localized in mPFC when forming impressions about individuals who appear friendly or threatening. Infants’ brain responses were measured using functional near-infrared spectroscopy while watching 4 different face identities displaying either smiles or frowns directed toward or away from them (*N* = 77). This was followed by a looking preference test for these face identities (now displaying a neutral expression) using eyetracking. Our results show that infants’ mPFC responses distinguish between smiling and frowning faces when directed at them and that these responses predicted their subsequent person preferences. This suggests that the mPFC is involved in impression formation in human infants, attesting to the early ontogenetic emergence of brain systems supporting person perception and adaptive behavior.

## Introduction

Humans are ultrasocial animals who live in complex groups (Tomasello 2014; Tomasello 2019). One of the most essential skills in navigating our social environments is our ability to learn to identify friendly (prosocial) individuals that we can affiliate and cooperate with and distinguish them from unfriendly or even hostile (antisocial) individuals that we may want to avoid (Fiske and Taylor 1991; Tomasello 2020). This ability for social evaluation or impression formation is considered to have deep evolutionary and ontogenetic roots, as it is shared with other primates and develops early during infancy (Hamlin et al. 2007; Wynn 2008; Wynn and Bloom 2014). In studies using functional magnetic resonance imaging (fMRI) with adults, the medial prefrontal cortex (mPFC) has been consistently identified as a key brain region involved in person perception and impressionformation (Mitchell et al. 2005; Mitchell et al. 2006; Wagner et al. 2012; Mende-Siedlecki et al. 2013). For example, in one of the first studies on this topic by Mitchell et al. (2005), participants were instructed to form impressions of people or objects on the basis of descriptions provided in an fMRI paradigm (i.e., this person “promised not to smoke in his apartment since his roommate was trying to quit,” or this car “recently had new fog lights installed”). In this study, adults displayed heightened mPFC activity when forming impressions about people but not when reasoning about objects (for reviews, see Amodio and Frith 2006; Ames et al. 2011). To date, little is known about the brain basis of infants’ early developing ability to form social impressions. Elucidating the brain processes involved in person perception and impression formation in infancy sheds light on the neurodevelopmental origins of this fundamental aspect of human social cognition.

Behavioral research attests that human infants possess the ability to form social impressions. For example, in seminal behavioral work, infants show a preference for helping agents and an avoidance of hindering agents, simply on the basis of observing third-party interactions between nonhuman characters, an ability thought to serve as the developmental foundation for morality (Hamlin et al. 2007; Wynn 2008; Wynn and Bloom 2014). This line of work has also inspired emerging research concerned with examining the neural correlates of impression formation and implicit moral evaluation using electroencephalography (EEG)-based methods (Cowell and Decety 2015a, 2015b). In 1 study, 1–2-year-olds’ visual preference for a helping agent over a hindering agent was predicted by brain responses evoked over frontal electrodes linked to processes of attentional and behavioral control (Cowell and Decety 2015b). The existing neurodevelopmental research on social impression formation is limited as it relies on older infants’ observation of third-party interactions of nonhuman characters and EEG measures, which do not provide direct insight into the brain regions involved in impression formation during social interaction in early ontogeny. The current study is aimed at overcoming these limitations by 1) examining impression formation while viewing other humans displaying social interactive facial signals, and 2) measuring localized brain responses using functional near-infrared spectroscopy (fNIRS).

To enable impression formation during social encounters with unknown individuals, preverbal infants may rely on tracking intentional and emotional facial cues to inform their person preferences and related approach and avoidance behaviors. For instance, seeing a social partner establish eye contact and smile might serve as an affiliative signal, whereas seeing someone frown during eye contact may serve as a threat signal (Niedenthal et al. 2010; Grossmann 2017). Previous work shows that preverbal infants detect eye contact and are also able to discriminate between smiles and frowns (Grossmann 2012; Krol et al. 2015; Grossmann 2017). This research suggests that, at least by 7 months of age, infants possess the social-perceptual skills to detect cues relevant for discerning whether another person’s face signals a friendly approach or imminent threat. However, it is unclear whether infants use this kind of information gleaned during social interactions to form impressions about individuals, guiding future behavior, and person preferences.

From a developmental brain perspective, prior research shows that infants employ both posterior superior temporal and medial prefrontal brain regions when processing emotional and gaze cues (Grossmann et al. 2008; Grossmann 2015). It is possible that mPFC engagement plays a more specific role in infants’ impression formation and coding for person preferences, as it has not only been shown to be involved during eye contact and smiling with an unfamiliar social partner (Grossmann et al. 2008), but it is further enhanced when infants view maternal smiles (Minagawa-Kawai et al. 2009). Moreover, as outlined above, research with adults assigns a specific role to the mPFC in impression formation and person perception (Amodio and Frith 2006).

**Figure 1:**
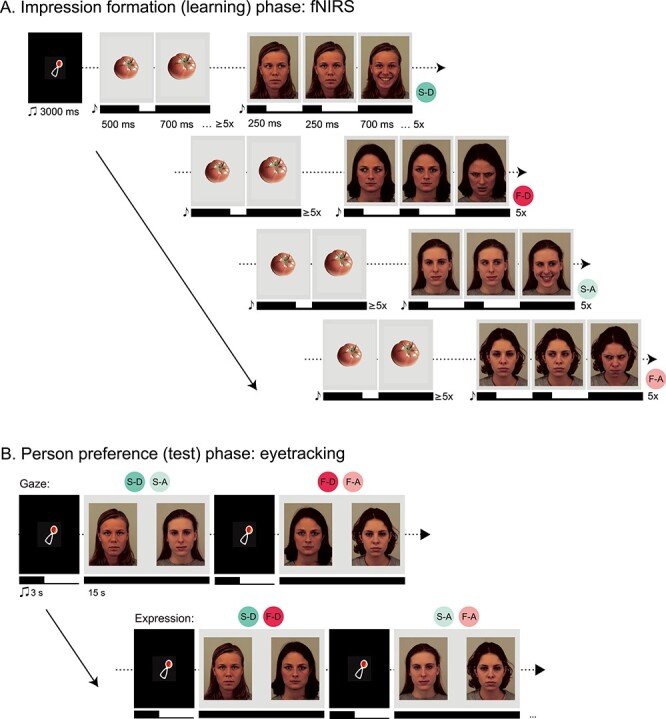
Full experimental paradigm. (*A*) “Impression formation (learning) phase.” Infants first viewed blocks of dynamic presentation of 4 identities shifting their gaze (from averted [A] to direct [D], or direct to averted) and subsequently presenting a facial expression (smile [S] or frown [F]), for a total of 4 possible expression-gaze combinations per block while fNIRS was recorded. Each trial began with a dynamic baseline consisting of nonsocial (vegetable) stimuli for at least 6 s, followed by a facial stimulus presentation for 6 s. Both baseline and facial stimulus presentation were preceded by a bell tone to maintain infant attention. Blocks were preceded by a 3 s audiovisual attention-getter in the center of the screen. (B) “Person preference (test) phase.” After fNIRS recording, infants immediately underwent a looking preference paradigm using eyetracking. Infants viewed static pairings of the 4 identities from the fNIRS paradigm; now presenting neutral, direct-gaze expressions for 15 s each. Each trial began with a 3 s audiovisual attention-getter in the center of the screen. Pairings were created to directly compare preferences for gaze (i.e., direct vs. averted-gaze within the 2 individuals who smiled [S-D vs. S-A]) and expression (i.e., smile vs. frown within the 2 individuals who exhibited direct gaze [S-D vs. F-D]) for a total of 4 pairings, each presented twice. Note that infants viewed photographs from the FACES database (35), but due to copyright restrictions we have recreated the stimuli using the publicly available Karolinska Directed Emotional Faces (KDEF) database (www.kdef.se); (ms = milliseconds, s = seconds, x = times).

The current study examined the neurodevelopmental origins of impression formation by testing the hypothesis that infants rely on processes localized in mPFC when forming person preferences of individuals that appear friendly or threatening. We measured infants’ brain responses using fNIRS while engaging them in an experimental setting approximating face-to-face interaction scenarios. Infants watched 4 different individuals (face identities) displaying a pseudo-dynamic shift of gaze (direct or averted-gaze) combined with 1 of 2 emotional expressions (a smile or a frown). Following this impression formation (learning) phase, infants’ person preference (viewing face identities holding a neutral expression) was assessed in a looking preference test using eyetracking (Fig. 1*A* and *B*). Our main analysis focused on brain responses in mPFC based on prior work implicating this region in impression formation and person perception in adults (Amodio and Frith 2006). We predicted that: 1) infants’ mPFC responses during the impression formation phase will distinguish between smiling and frowning individuals with a direct gaze and 2) mPFC responses will correlate with infants’ person preferences during the test phase. In order to test for the specificity of infants’ mPFC involvement during impression formation, we assessed infants’ brain responses in additional brain regions: the posterior superior temporal cortex (pSTC) and the temporoparietal junction (TPJ), both regions previously shown to be involved in social perception and cognition (Hoffman and Haxby 2000; Pelphrey et al. 2004; Decety and Lamm 2007; Chiao, Harada et al. 2009; Bzdok et al. 2016; Igelstrom and Graziano 2017; Cacioppo et al. 2018; Hyde et al. 2018). In particular, prior work using fNIRS with infants has implicated the pSTC in eye gaze processing during face-to-face interactions (Grossmann et al. 2008) and the TPJ in theory of mind (Hyde et al. 2018). Taken together, this novel experimental paradigm allowed for the systematic investigation of impression formation in the infant’s brain and its link to behavior reflected in looking preferences.

## Materials and Methods

### Participants

Seventy-seven typically developing 11-month-old infants participated in this study (*M*_age_ = 339.84 days, *SD* = 6.77; 36 females). All infants were born at a normal birth weight (>2500 grams) and standard gestational age (>38 weeks). Infants were of parent-reported European descent and resided in the city of Leipzig, Germany, which is a metropolitan area with a population of about 570 000 people and a comparably low rate (13.3%) of people with an immigrant background, primarily from other European countries. Mothers reported an average of 17.02 years of education (ranging from 10 to 24 years), and 85.7% were still on maternity leave at the time of testing. An additional 20 infants were tested, but excluded from analyses based on an a priori exclusion criterion of at least 2 artifact-free trials per fNIRS condition. The minimum sample size was partly determined based on a literature review of comparable infant neuroimaging and eyetracking designs (Peltola et al. 2009; Rajhans et al. 2016; Jessen and Grossmann 2017; Grossmann et al. 2018; Peltola et al. 2020) but chosen to be much larger than in mentioned prior studies in order to strengthen the confidence in the obtained findings. This study was approved by the Ethics Committee at the Medical Faculty, Leipzig University (236-10-23082010) and was conducted in accordance with the Declaration of Helsinki. Parents provided written informed consent and were compensated with travel money, a toy for the infant, and a printed photograph of their infant in the fNIRS cap.

### Stimuli

Color photographs of Caucasian females with direct-gaze expressions of happiness (displaying smiles), anger (displaying frowns), and neutrality were chosen from a validated stimulus set (FACES Collection) (Ebner et al. 2010). Four actress identities were selected based on expression recognition rates by groups of young, middle-aged, and older adults as well as on the basis of minimal distracting features (i.e., hair was not obstructing face). Average expression recognition accuracy within the 4 selected identities was over 94.92% (see Ebner et al. 2010). The eye gaze was manipulated using Adobe Photoshop CS5 for use in the fNIRS paradigm (Fig. 1*A*). Photographs were resized and cropped to align with fixed markers for the position of the 2 eyes, mouth, and nose in the center of a gray background. This editing technique ensured that all facial features were presented in the same location on the screen. Baseline images consisted of color photographs of 4 inanimate objects (vegetables) presented in the center of the same light gray background. These images have been successfully used as baseline stimuli in fNIRS studies of face processing in infants (Otsuka et al. 2007; Nakato et al. 2009; Nakato et al. 2011; Grossmann et al. 2018; Krol et al. 2019). For the eyetracking procedure, photographs of the actresses with a direct gaze and neutral facial expressions were presented side by side on the same gray background (Fig. 1*B*).

### Procedure

Experiments consisted of the fNIRS paradigm (impression formation learning phase) immediately followed by the eyetracking paradigm (person preference test). Infants were seated on a parent’s lap in a quiet, dimly lit room, facing a screen (52 cm × 32 cm) at a distance of approximately 60 cm. A room divider separated the experimental area from the control desk, and a black curtain covered the region behind the presentation monitor in order to prevent distractions. As in prior studies (Grossmann et al. 2018; Krol et al. 2019), a plastic ring attached to the chair was provided for each infant to hold in order to reduce arm and body movements. A camera was attached to the bottom of the screen for online tracking of infant behavior as well as offline coding of attention to each trial. After measurement of head circumference, infants were fitted with an appropriately-sized custom-built elastic cap that held the NIRS probes (detailed below). Photographs were taken of the front and side head placement of the cap for future reference.

The fNIRS paradigm consisted of a total of 14 blocks, each containing a randomized presentation of the 4 trial-types: smiling-direct, smiling-averted, frowning-direct, and frowning-averted facial expressions. Therefore, each infant had the possibility to view 56 trials (14 per condition). Critically, each of the 4 identities consistently presented the same expression-gaze combination throughout the experiment. Different identity-expression-gaze combination scenarios were created to account for all 24 possible combinations and were counterbalanced across infants. That is, identity-expression-gaze combinations remained constant within subjects, but not between, and this was to account for potential identity effects. Each presentation block began with an attention-getter to keep infants alert and to orient them to the center of the screen (a shaking rattle, as described in Krol et al. 2015). Each trial began with the presentation of a baseline stimulus for at least 6 s followed by a face stimulus for 6 s. At the beginning of each baseline and face presentation (twice per trial), a brief 150 ms bell tone (about 600 Hz) sounded to maintain infant attention. Baseline and face stimuli were presented dynamically. The baseline shifted from an image changing from its original size (500 ms) to a slightly larger size (~1° increase in visual angle) (700 ms) at least 5 times. Face presentation consisted of 3 photographs of the same identity: 1) a neutral expression with the nontarget gaze (250 ms), 2) a neutral expression with the target gaze (250 ms), and finally, 3) the target expression (smile or frown) with the target gaze (direct or averted) (700 ms). This sequence repeated 5 times to create the illusion of a neutral individual first shifting their gaze and subsequently shifting from a neutral expression to either a smile or frown (Fig. 1*A*). This method of pseudo-dynamic presentation of facial expressions was adapted from previous infant fNIRS paradigms (Nakato et al. 2011; Grossmann et al. 2018; Krol et al. 2019) and ensured that infants maintained attention during the long trials that fNIRS measurement requires. Stimulus presentation was counterbalanced such that no expression or gaze trial type was shown more than twice in succession. Infants were shown an average of 25.65 total fNIRS trials (range = 10 to 46; *SD* = 7.57).

**Figure 2:**
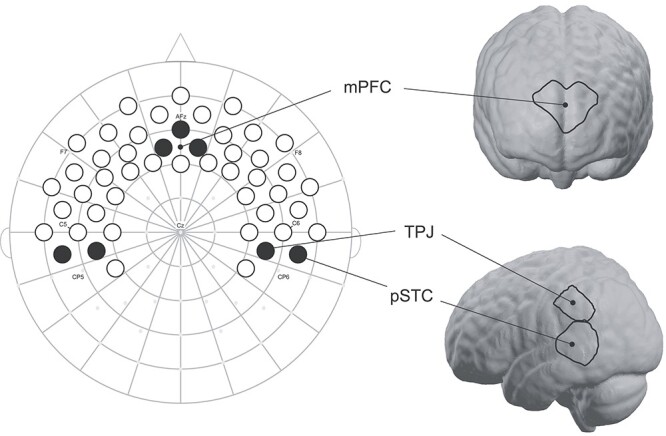
fNIRS cap template and ROIs. Shown is our fNIRS cap template mapped onto 10–20 space. International 10-20 coordinates are indicated by small gray dots, and relevant coordinates are labeled. Forty-nine channels (source-detector pairs) are presented as circles; those shaded in black are the channels used to create ROIs for mPFC, bilateral TPJ, and bilateral posterior superior temporal cortices (pSTC). Channels have been projected onto MNI brain space using NIRSite and nirsLAB software (NIRx) for readers’ reference.

Stimuli were presented using Presentation software (Neurobehavioral Systems, MA), and fNIRS data were recorded using a NIRScout system and NIRStar acquisition software (NIRx, Berlin, Germany). Hemoglobin absorption was measured using 32 optodes (16 sources, 16 detectors) placed at approximately 2 cm distance over frontal and temporal cortices on a custom-built elastic cap (EasyCap, Germany) with standard 10–20 references. This arrangement comprised 49 channels (source-detector pairs) from which to glean hemodynamic activity (see Fig. 2 for fNIRS cap template). Prior to each recording, channels were calibrated within NIRStar. During calibration, signal qualities of each channel were updated in real-time to reflect current gain and corresponding noise, and were classified into 4 color-coded groups: excellent, acceptable, critical, and lost. We did not begin recordings unless all channels showed excellent or acceptable signal qualities. The color-mapped channel calibrations for each participant were saved for reference prior to analyses. Data were recorded at a sampling rate of 6.25 Hz. Near-infrared light was emitted at 2 wavelengths (760 nm and 850 nm) with a power of 5 nm/wavelength. The system automatically adjusted light intensity in order to provide optimal gain.

Upon completion of the fNIRS paradigm, caps were removed and infants immediately underwent the eyetracking paradigm, lasting approximately 3 min. Stimuli were presented with Tobii Studio 3.2 (Danderyd, Sweden) and obtained with a Tobii X120 eyetracker, which has a sampling rate of 120 Hz and collects information from both eyes. 5-point calibration was conducted prior to stimulus presentation using the same methods as reported in our previous work (Krol et al. 2015), and involved a shaking rattle stimulus occurring sequentially within the 4 corners and center of the screen. The rattle had a visual angle of approximately 4.6° and was paired with a sound to initiate attention. Calibration was deemed successful when the infant fixated within all 5 locations and was repeated until this occurred. Detailed information on the infant calibration procedure within Tobii Studio is located in the Tobii user manual (www.tobii.com). As detailed in Figure 1*B*, trials were constructed as a side-by-side presentation of 2 identities for 15 s, paired to directly compare between gaze (i.e., the 2 actresses who smiled [direct vs. averted]) and expression (i.e., the 2 actresses who presented direct gaze expressions [smile vs. frown]). Thus, a total of 4 pairings were presented. Each pairing was presented twice such that for each pair of identities, each respective identity was presented once on each side of the screen, for a total of 8 trials. Each trial was preceded by the same audiovisual attention-getter as presented in the fNIRS paradigm to maintain and orient attention to the center of the screen.

### fNIRS Analysis

Videos from each session were manually coded for infant looking duration to each trial. Trials were only included if infants attended to the screen at least 4 of the 6 s for which both baseline and face stimuli were presented. The fNIRS data were then visually inspected for motion artifacts. Trials with motion artifacts were removed from further analyses. The remaining data were analyzed using the MATLAB-based software Nilab2 (NIRx, Germany). Data were filtered with a 0.2-Hz low-pass filter in order to remove fluctuations due to infant heart rate and a high-pass filter of 0.083 Hz (12 s) to remove changes too slow to be related to experimental stimuli (i.e., fluctuations due to drift). Measurements were converted into oxygenated hemoglobin (oxy-Hb) and deoxygenated hemoglobin (deoxy-Hb) using the modified Beer–Lambert law. Wavelength-specific differential pathlength factors of 7.25 (760 nm) and 6.38 (850 nm) were used, as suggested by NIRx (Essenpreis et al. 1993; Kohl et al. 1998; Zhao et al. 2002). Boxcar functions corresponding to the 4 stimulus conditions were convolved with a standard hemodynamic response function based on the stimulus length parameter (Boynton et al. 1996). Peak response was set to 5 s, in line with previous reports from vascular imaging in infants (Taga et al. 2003; Homae et al. 2006). The average concentration changes of oxy-Hb and deoxy-Hb in response to each stimulus condition (from baseline) were extracted for each channel, for each individual infant. Only infants who provided at least 2 artifact-free trials per condition were included in fNIRS analyses. Out of 97, 20 infants were excluded on the basis of this criterion, resulting in a final fNIRS sample size of *N* = 77.

Regions of interest (ROIs) containing the mPFC, bilateral pSTC, and bilateral TPJ were created by referencing anatomical sources of the infant 10–20 system (Kabdebon et al. 2014) and through the use of nirsLAB and NIRSite software (NIRx), which projects fNIRS channels onto standard adult MNI space (locked to 10–20 coordinates) (see Fig. 2). In addition to analyzing average concentration change for each of the 4 conditions, a difference score encompassing all 4 conditions was computed to assess brain response bias to the smiling, direct-gaze face as compared to the response to the frowning, direct-gaze face, while accounting for the response to the averted-gaze faces as follows:

$$\begin{eqnarray*} \mathrm{Brain}\ {\mathrm{response}\ \mathrm{bias}}_{\mathrm{ROI}}&=&\left({\mathrm{OxyHbSmileDirect}}_{\mathrm{ROI}}\right.\nonumber\\ &&\left.-\mathrm{OxyHbSmileAverte}{\mathrm{d}}_{\mathrm{ROI}}\right)\nonumber\\ &&-\left({\mathrm{OxyHbFrownDirect}}_{\mathrm{ROI}}\right.\nonumber\\ &&\left.-\mathrm{OxyHbFrownAverte}{\mathrm{d}}_{\mathrm{ROI}}\right) \end{eqnarray*}$$

Brain response bias scores were calculated for each ROI separately. Scores above zero indicate increased brain response bias to the smiling, direct-gaze face, while below zero indicate increased brain response bias to the frowning direct-gaze face.

### Eyetracking Analysis

Creation of areas of interest (AOI) and extraction of data occurred within Tobii Studio. AOIs comprising the eye region of each facial stimulus were created (Supplemental Figure S1). The decision to consider the eye region, as compared to the full face, was based on prior work suggesting that infants would use direct eye contact as a signal for the desire to communicate (Argyle and Dean 1965; Ho et al. 2015; Canigueral and Hamilton 2019). Additionally, the inclusion of looking to the rest of the face (and thus potential avoidance of eyes) may have actually captured aversion to, rather than a preference for, a particular identity (Csibra 2010). Tobii Studio automatically filters out invalid or missing data prior to computing looking duration (i.e., in the case of a blink, both eyes would register as missing data and would thus be removed). Note that prior to data extraction, we visualized heat maps of each attention-getter trial, per infant, to check for potential drift across the sessions as well as to confirm central looking to the screen prior to trial onset. The total looking duration to the eye region of each individual was extracted per infant for each trial type. In order to assess preference for particular identities, looking bias scores were computed by calculating the proportion of looking time to each individual. For example, in order to assess a bias for direct versus averted-gaze within the 2 identities who smiled:

$$\begin{eqnarray*} \mathrm{DirectGazeBia}{\mathrm{s}}_{\mathrm{Smile}}&=&\frac{\mathrm{Total}\ \mathrm{looking}\ \mathrm{duratio}{\mathrm{n}}_{\mathrm{Smile}\mathrm{Direct}}}{\left(\mathrm{Total}\ \mathrm{looking}\ \mathrm{duratio}{\mathrm{n}}_{\mathrm{Smile}\mathrm{Direct}}\right.}\nonumber\\ &&{\left.+\mathrm{Total}\ \mathrm{looking}\ \mathrm{duratio}{\mathrm{n}}_{\mathrm{Smile}\mathrm{Averted}}\right)} \end{eqnarray*}$$

Altogether, this yielded 4 looking bias scores for each infant: 1) “SmileExpressionBias_Direct_”: expression preference for the smiling, direct-gaze individual as compared to the frowning, direct-gaze individual; 2) “SmileExpressionBias_Averted_”: expression preference for the smiling, averted-gaze individual as compared to the frowning, averted-gaze individual; 3) “DirectGazeBias_Smile”_: gaze preference for the direct-gaze, smiling individual as compared to the averted-gaze, smiling individual; and 4) “DirectGazeBias_Frown_”: gaze preference for the direct-gaze, frowning individual as compared to the averted-gaze, frowning individual. Similar to the above fNIRS analysis, we again calculated a global bias (difference) score that encompassed all eyetracking variables, in which higher values indicate increased attentional allocation to the identity that presented a happy, direct-gaze expression:

$$ \mathrm{Person}\ \mathrm{preference}=\mathrm{DirectGazeBia}{\mathrm{s}}_{\mathrm{Smile}}-\mathrm{DirectGazeBia}{\mathrm{s}}_{\mathrm{Frown}} $$

Note that performance on the eyetracking task crucially depended on infants’ attention during the fNIRS task. We therefore only analyzed infants who exceeded fNIRS exclusion criteria, indicating that they successfully viewed at least 2 trials of each emotion-gaze combination. In addition, the average trials viewed per condition during fNIRS was included as a covariate in all eyetracking analyses to account for differing experience with and memory of the expression-gaze presentations.

## Results

### Infants’ Brain Responses in mPFC During Impression Formation Predict Person Preference

**Figure 3:**
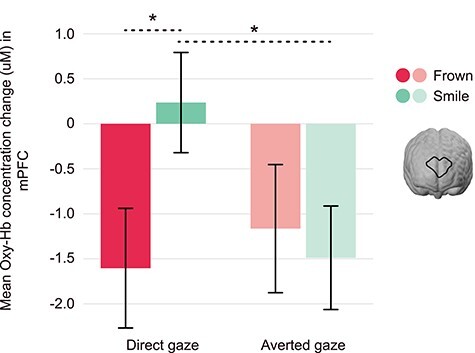
Infant mPFC is sensitive to frowning and smiling faces with direct and averted-gaze. Displayed is the interaction between expression (smile vs. frown) and gaze (direct vs. averted) in the mPFC, *F*(1, 76) = 4.68, *P* = 0.034, *ηp^2^* = 0.058, as indexed by the concentration change of oxygenated hemoglobin (oxy-Hb). The mPFC discriminates between frowning and smiling faces of direct gaze, as well as between direct and averted-gaze within smiling faces, displaying the highest response to smiling, direct-gaze faces; error bars represent standard error of the mean, ^*^P < 0.05 (uM = microMolar).

A 2 × 2 repeated-measures ANOVA was conducted to assess brain responses in the mPFC, with expression and gaze as within-subject factors. This revealed a significant interaction between expression and gaze, *F*(1, 76) = 4.68, *P* = 0.034, *ηp^2^* = 0.058 (Fig. 3). The mPFC response discriminated between frowning and smiling individuals exhibiting direct gaze, *F*(1, 76) = 4.30, *P* = 0.041, *ηp^2^* = 0.054. This analysis also showed that smiling, direct-gaze faces evoked greater brain responses than smiling averted-gaze faces, *F*(1, 76) = 4.61, *P* = 0.035, *ηp^2^* = 0.057. As an exploratory analysis, we repeated the above analysis with infant sex as a between-subjects factor. There was no main effect of infant sex, nor any interactions with sex (all *P*-values > 0.05).

**Figure 4:**
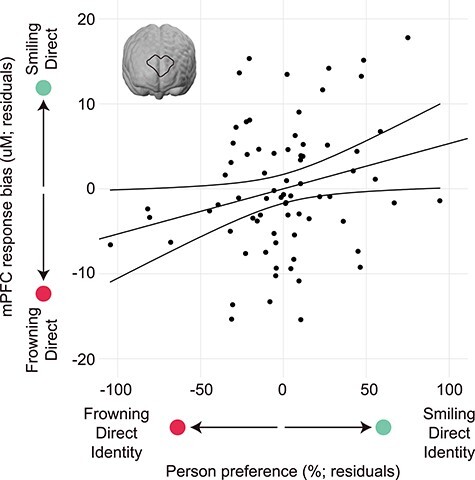
Infants’ mPFC response during impression formation predicts person preference. Plotted are the residuals from a regression demonstrating that heightened mPFC response bias for the smiling, direct-gaze face during impression formation predicts an increased looking preference for that identity in a looking preference paradigm (now displaying a neutral face). In contrast, a heightened mPFC response bias for the frowning, direct-gaze face predicts an increased looking preference for that identity (now displaying a neutral face), *β* = 2.50, *t* = 2.142, *P* = 0.036 (uM = microMolar).

Brain responses and looking preferences were transformed into bias (difference) scores encompassing all stimuli, such that scores above zero reflected increased brain response and/or preference for the individual presenting a smiling, direct-gaze expression, and scores below zero reflected increased brain response and/or preference for the individual presenting a frowning, direct-gaze expression (see Materials and Methods section). In order to test the hypothesis that brain response in the mPFC during the learning phase might predict later person preference, a multiple regression was performed with mPFC response bias and average trials viewed as predictors, and person preference during eyetracking as the dependent variable. This analysis revealed that the mPFC response during impression formation predicted an increased looking preference for that identity (*β* = 2.50, *t* = 2.142, *P* = 0.036) (Fig. 4). Specifically, enhanced mPFC recruitment while viewing the individual with the smiling, direct-gaze expression predicted an increased preference for that identity in the eyetracking paradigm, now displaying a neutral expression. In contrast, increased mPFC response to the individual presenting a frowning, direct-gaze expression predicted an increased preference for that identity in the eyetracking paradigm. This suggests that recruitment of the mPFC is involved in impression formation such that preference for a particular person’s face is predicted by greater recruitment of the mPFC during learning in the impression formation phase. For comparison purposes, we conducted 2 additional multiple regressions using bilateral pSTC and bilateral TPJ response bias as predictors. These analyses showed no predictive capacity of either the pSTC response or TPJ response on later person preference (all *P*-values > 0.05).

### Analysis of Infants’ Brain Responses in pSTC and TPJ During the Impression Formation Phase

**Figure 5:**
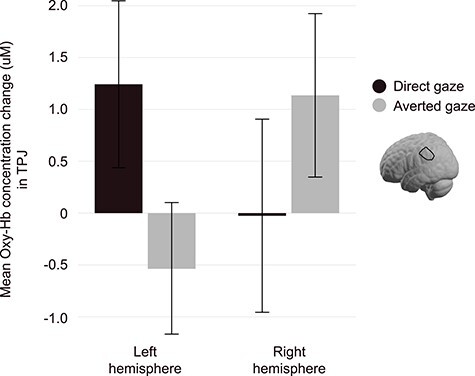
Infant TPJ is sensitive to direct and averted-gaze. Displayed is the interaction between hemisphere (left vs. right) and gaze (direct vs. averted) in the TPJ, *F*(1, 76) = 6.32, *P* = 0.014, *ηp^2^* = 0.077, as indexed by the concentration change of oxygenated hemoglobin (oxy-Hb). The TPJ better discriminates between direct and averted-gaze in the left hemisphere, displaying a heightened response to direct-gaze versus averted-gaze faces; error bars represent standard error of the mean (uM = microMolar).

Repeated-measures ANOVAs were performed for both pSTC and TPJ regions with hemisphere, expression, and gaze as within-subject factors. Our analysis of the pSTC did not reveal any main effects or interactions (all *P*-values > 0.05). In the TPJ, a hemisphere × gaze interaction was revealed (*F*(1, 76) = 6.32, *P* = 0.014, *ηp^2^* = 0.077) (Fig. 5). Post-hoc analyses suggest that the left hemisphere better discriminated between direct and averted-gaze faces, regardless of expression, with direct gaze evoking greater responses than averted-gaze (*F*(1, 76) = 3.44, *P* = 0.068); however, note that this was only marginally significant outside of the interaction. Including infant sex as a between-subjects factor in these analyses did not impact pSTC findings (no significant interactions or main effect of sex, all *P*-values < 0.05). When infant sex was included in the TPJ analysis, a hemisphere × expression × sex interaction emerged, *F*(1,75) = 4.072, *P* = 0.047, *ηp^2^* = 0.041. In both male and female infants, brain responses in TPJ were nearly identical across hemispheres when viewing smiling faces. When viewing frowning faces, however, female infants showed greater responses in the left hemisphere, whereas male infants showed greater responses in the right hemisphere.

### Exploratory Analyses of Person Preferences

The main goal of this study was to explore the capacity of infants’ mPFC response in predicting person preference assessed with eyetracking. However, to see whether group preferences for particular individuals might differ by expression or eye gaze, we conducted exploratory analyses on the eyetracking data alone. We predicted that infants may show a preference for those individuals who might signal a positive social interaction (the identities who previously smiled with direct-gaze), and a potential avoidance of identities who might signal impending threat (those who previously frowned with direct-gaze). Repeated-measures ANCOVAs were conducted on eyetracking bias scores to see 1) whether infants’ preference for direct versus averted-gaze identities was impacted by the expression presented (smile vs. frown), and 2) whether infants’ preference for smiling versus frowning identities was impacted by the “gaze shift” presented (direct vs. averted). The average number of trials viewed during impression formation was included as a covariate. On the group level, no evidence was found for an impact of expression on preference for direct-gaze versus averted-gaze identities, *P* > 0.05. Similarly, there was no impact of gaze shift on preference for smiling versus frowning identities, *P* > 0.05. We repeated these analyses with infant sex as a between-subjects factor. This analysis revealed an expression × sex interaction on infant gaze preference, (*F*(1, 72) = 4.135, *P* = 0.046, *ηp^2^* = 0.054)). Male infants’ preference for direct- versus averted-gaze identities did not differ by expression. In contrast, female infants’ preference for direct- versus averted-gaze identities differed by the expression presented, in the predicted pattern (Supplemental Figure S2). Female infants showed a preference for direct-gaze identities who smiled, and they also showed an avoidance of direct-gaze identities who frowned.

## Discussion

This study examined the neurodevelopmental origins of impression formation and its link to person perception in infancy. Confirming our hypothesis, the current results show that infants rely on processes localized in mPFC when forming an impression and evaluating previously unknown individuals during social interactions based on their nonverbal behavior. Specifically, our findings show that: 1) infants’ mPFC responses distinguish between smiling and frowning individuals during eye contact, and 2) mPFC responses during impression formation predicted infants’ subsequently measured person preference. These findings demonstrate that the mPFC is involved in impression formation in human infants, providing evidence that the brain system supporting person perception develops early in human ontogeny. This suggests that the ability to form impressions during social encounters may represent a foundational element of the human social-cognitive make up, supporting adaptive behavior.

From a brain science perspective, the current results add to an emerging body of work with infants providing evidence that mPFC function plays a role in early social cognition and may guide learning from and about others (Grossmann 2013). Our findings also contribute to closing the gap between the extensive behavioral work showing sophisticated social-cognitive skills in infancy (Spelke and Kinzler 2007; Woodward 2009; Baillargeon, Scott et al. 2010) and social neuroscience work with adults, highlighting mPFC as a key brain region involved in complex social-cognitive processes afforded by engaging with and thinking about others (Amodio and Frith 2006; Frith and Frith 2006). More specifically, our fNIRS results show that infants’ mPFC integrates information about another person’s gaze direction and emotional expression during face-to-face interactions as shown in the enhanced response in this brain region seen specifically to direct gaze smiles. This replicates previous work using fNIRS with infants showing enhanced mPFC responses to direct gaze smiles (Grossmann et al. 2008) and critically extends it by demonstrating that it is specific to smiles and not seen in response to other emotional expressions, namely frowns or threats. This suggests that an interactive partner who makes eye contact and smiles may play a privileged role in recruiting infants’ mPFC, which might be explained by infants viewing direct gaze smiles as affiliative signals (Csibra and Gergely 2009; Niedenthal et al. 2010), capable of promoting trust and cooperation (Stallen and Sanfey 2013).

Our results further show that infants’ mPFC responses during face-to-face interactions in the impression formation phase predict their person preference shown during a test phase measuring looking time. Specifically, a greater response bias in mPFC for a particular expression-gaze combination during the learning phase predicted a greater preference for that particular person during the test phase. This suggests that infants’ mPFC is involved in the formation of person preferences based on emotional signaling. In this context, it is important to emphasize that this predictive effect of mPFC responsivity was seen in the absence of emotional cues during the test phase as all faces displayed neutral expressions, indicating that infants effectively used and transferred the learned impression about a person to inform their subsequent looking behavior. It is also worth noting that the current results indicate that infants are able to keep track of and learn about 4 different individuals—face identities—during the impression formation phase, which attests to their competence in learning and using the information in socially complex contexts.

The current results supporting a brain-behavior correlation also show that there is variability between infants in terms of the direction of the effect or preference for a particular person associated with smiling or frowning at them. In other words, as seen in Figure 4, while some infants display greater differential brain and behavioral responses to a person associated with direct gaze smiles, other infants display greater differential brain and behavioral responses to a person associated with direct gaze frowns. This finding is in line with previous reports from older infants linking EEG responses when observing prosocial versus antisocial third-party interactions between nonhuman animated characters to subsequent looking preferences for these agents, which showed a similar pattern of variability among infants (Cowell and Decety 2015). Moreover, when considering the eyetracking results at the group level, we did not obtain a clear looking preference for any particular individuals on the basis of their previously shown emotional expression or eye gaze direction. This finding is directly in line with findings from a previous study (Cowell and Decety 2015), in which 12- to 24-month-old infants were shown helping or hindering behavior by animated characters while EEG was recorded. In this study, after viewing helping and hindering behaviors, infants showed no clear preference for helpers versus hinderers; instead, similar to our findings, infants’ electric brain responses while viewing moral characters predicted their behavioral preferences.

In an additional exploratory analysis using sex as a between-subjects variable, we found the predicted person preferences in female infants but not in male infants. Specifically, female infants demonstrated a looking preference for the individual who smiled with a direct gaze and an avoidance of the individual who frowned with a direct gaze. Male infants showed no differentiation in their preferences for smiling and frowning individuals. Importantly, male and female infants showed no difference in mPFC response during impression formation, suggesting similar brain sensitivity when detecting emotional and gaze cues across sex. Instead, the obtained behavioral preference effect suggests that the female infants in the current study might have been more effective at forming adaptive preferences reflected in greater attention to the affiliative person (the smiling, direct-gaze individual) and greater avoidance of the threatening person (the frowning, direct-gaze individual). A host of studies index sex differences in various aspects of social cognition, including emotion recognition, theory of mind, and empathy; generally suggesting a female advantage in both children and adults (McClure 2000; Baron-Cohen and Wheelwright 2004; Calero, Salles et al. 2013; Ibanez, Huepe et al. 2013). It nonetheless seems premature to draw any strong conclusions from the findings of our exploratory analysis, but clearly, future research is needed to examine factors such as sex contributing to individual differences in infants’ person perception and preferences. Based on previous work, other factors that may contribute to variability in infants’ social preferences may include both intrinsic factors such as genetic variability in the oxytocin system, and extrinsic factors such as caregiver behavior and values (Cowell and Decety 2015; Krol et al. 2015).

More generally, it seems important to discuss the potential mechanisms accounting for infants’ impression formation abilities displayed in the current study. One may contest our interpretation of infants using rather sophisticated social-cognitive processes in this task by arguing that the effects obtained can be explained by more basic, unspecific associative learning, whereby infants simply associate visual stimuli with negative or positive experiences. While we agree that learning by association plays a role here, it cannot fully account for infants’ responses. First, the current data show that infants use similar brain processes as adults localized in mPFC, which show a high degree of specificity for processing social and intentional information in adults (Amodio and Frith 2006), rendering it unlikely that the observed effects are driven by general associative learning. In fact, recent work using fMRI with infants shows a similar degree of social specificity as infant mPFC involvement is only seen in response to dynamic faces but not to other nonsocial visual scenes (Deen et al. 2017, Powell et al. 2018). Second, if the mere association with positive and negative experiences were to account for person preferences, then discriminatory effects between smiling and frowning individuals should occur independent of gaze direction. Yet, our data show that this is only the case during eye contact. Third, our results also show that another brain region—the TPJ—previously shown to be involved in theory of mind processing in infants (Hyde et al. 2018), shows differential responses on the basis of the direction of the gaze shift displayed by the individual with enhanced responses during eye contact. However, infants’ responses in this brain region were not sensitive to the emotional expressions displayed by the person and also did not relate to infants’ person preference, further supporting the notion of specificity in the mPFC’s involvement in impression formation. Taken together, our results, therefore, suggest that infants rely upon specific brain processes in interpreting and learning from facial signals relevant to the self.

There are a few methodological considerations that warrant further discussion. First, we did not find an impact of the target gaze on infants’ pSTC responses as previously reported in studies with infants and adults (Hoffman and Haxby 2000; Pelphrey et al. 2004; Grossmann et al. 2008). It is possible that this is due to differences in study design across studies. In particular, prior studies were specifically designed to examine the contrast conditions that differed in the direction of gaze, whereas the current study consisted of a pseudo-dynamic gaze shift from direct to averted (or vice versa), repeating 5 times. Therefore, in the current study, each trial contained exemplars of both direct and averted-gaze, making it difficult to compare it directly to prior work which manipulated gaze direction in separate experimental conditions. Second, an inherent limitation of fNIRS is that it does not have the resolution to capture brain activity from deeper cortical and subcortical brain regions (Lloyd-Fox et al. 2010). With respect to the current study, it is thus unlikely that the measured fNIRS responses capture activity from all the portions of the mPFC that can be imaged with fMRI, especially when considering those located deeper on the medial wall. However, some evidence exists from research with adults comparing fMRI and fNIRS responses indicating a strong correlation between mPFC responses seen in fMRI and fNIRS including activity seen in the dorsomedial wall along the longitudinal fissure (Sasai, Homae et al. 2012). Notably, in this context, adult fMRI research on person perception and impression formation with adults most frequently reports a specific involvement of dorsal rather than ventral parts of the mPFC (Wagner et al. 2012). Another methodological limitation concerns the adequacy of using eyetracking (and looking time) to examine person preferences in infants, as it may lack ecological validity and not directly tap into overt behavioral preferences. More specifically, the current results from a looking preference paradigm, while providing excellent experimental control of the stimulus material, cannot be easily compared to real-world social interaction scenarios that provide infants with a wealth of behavioral cues to guide person perception and shape their impression of others. Future research would benefit from utilizing live interaction partners and behavioral designs that further examine infant person preferences (i.e. determine whether infants will help or play with particular individuals more than others) (Kuhlmeier et al. 2014). For example, one could imagine a live interactive study carried out in conjunction with portable fNIRS recording.

In conclusion, the current findings show that the brain systems supporting the essential social skills of forming impressions about others and distinguishing between friendly and hostile individuals develop early in human ontogeny. This provides neurodevelopmental evidence for theories stipulating that humans are adapted to live in complex social environments and possess vital cognitive skills that enable affiliation, group life, and cooperation.

## Supplementary Material

Krol_CCC_2020AcceptedSuppMaterial_tgaa070
