# Supplementary material for: Impression Formation in the Human Infant Brain
Source: Cereb Cortex Commun. 2020 Sep 29;1(1):tgaa070. doi: 10.1093/texcom/tgaa070 (PMC7592636; doi:10.1093/texcom/tgaa070)
Supplement: Krol_CCC_2020AcceptedSuppMaterial_tgaa070 [file krol_ccc_2020acceptedsuppmaterial_tgaa070.zip › Krol_CCC_2020AcceptedSuppMaterial_tgaa070.docx]

**Supplementary Material**


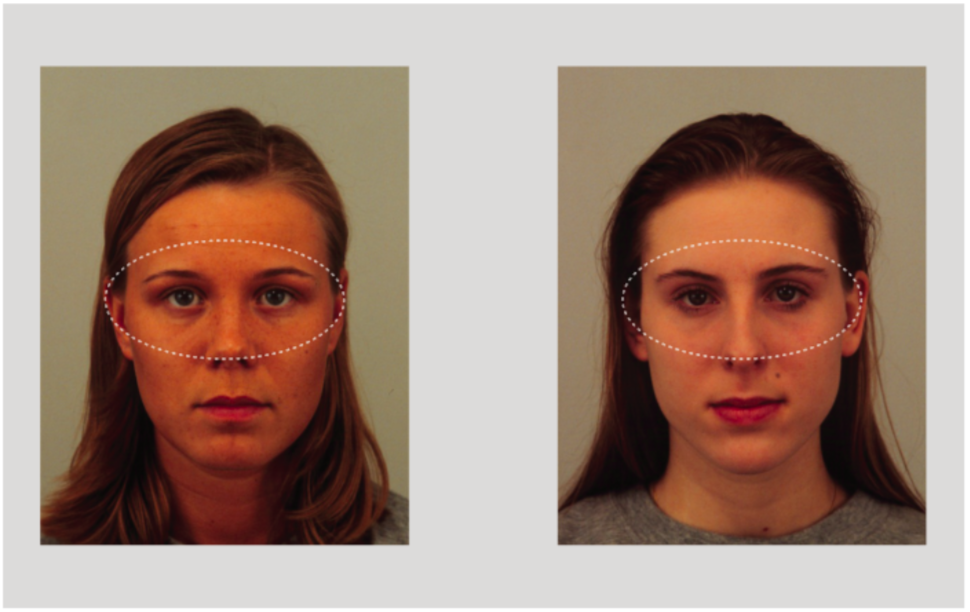


**Supplemental Figure 1. Eyetracking areas of interest (AOIs).** AOIs comprised an ovular region encompassing the eyes of each identity for person preference analysis. Please note that infants viewed photographs from the FACES database (35), but due to copyright restrictions we have recreated the stimuli using the publicly available Karolinska Directed Emotional Faces (KDEF) database ([www.kdef.se](http://www.kdef.se)).


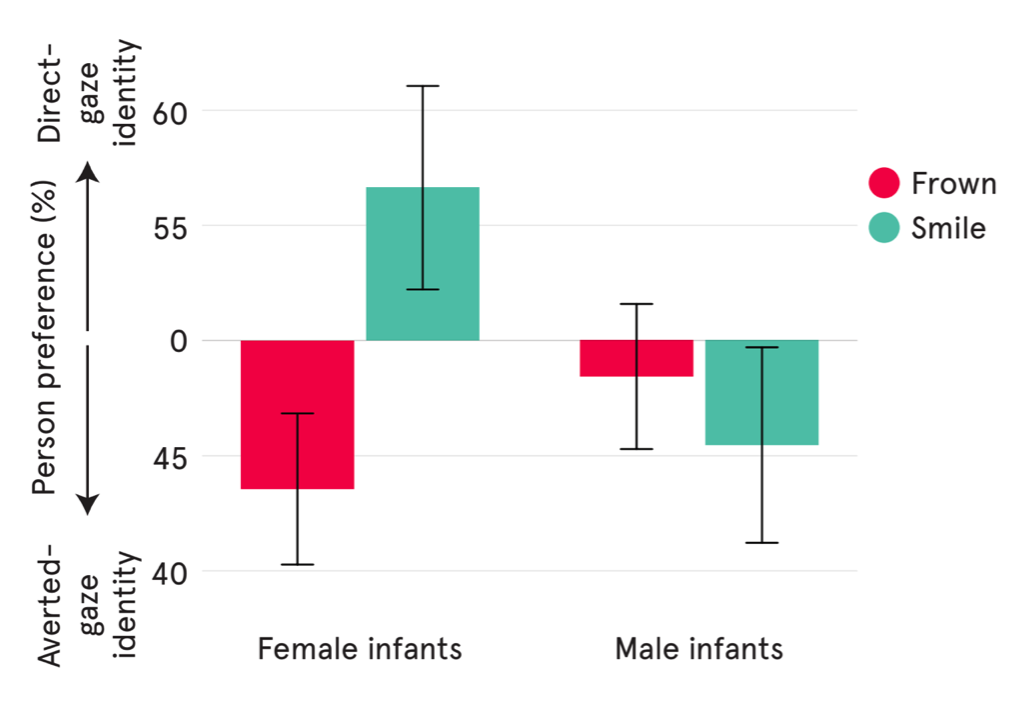


**Supplemental Figure 2. Infant gaze preference for identities who previously frowned or smiled is impacted by sex.** Displayed is the interaction between expression (frown vs. smile) and sex on infant preferences for direct vs. averted gaze identities, *F*(1, 72) = 4.14, *p* = 0.046, *ηp^2^* = 0.054. Male infants’ preference for direct- vs. averted-gaze identities did not differ by expression. In contrast, female infants’ preference for direct- vs. averted-gaze identities differed by the expression presented. Female infants showed a preference for direct-gaze identities who smiled, and showed an avoidance for direct-gaze identities who frowned; error bars represent standard error of the mean.
